# *C6orf10* Low-Frequency and Rare Variants in Italian Multiple Sclerosis Patients

**DOI:** 10.3389/fgene.2019.00573

**Published:** 2019-06-26

**Authors:** Nicole Ziliotto, Giovanna Marchetti, Chiara Scapoli, Matteo Bovolenta, Silvia Meneghetti, Andrea Benazzo, Barbara Lunghi, Dario Balestra, Lorenza Anna Laino, Nicolò Bozzini, Irene Guidi, Fabrizio Salvi, Sofia Straudi, Donato Gemmati, Erica Menegatti, Paolo Zamboni, Francesco Bernardi

**Affiliations:** ^1^Department of Life Sciences and Biotechnology, University of Ferrara, Ferrara, Italy; ^2^Department of Biomedical and Specialty Surgical Sciences, University of Ferrara, Ferrara, Italy; ^3^IRCCS Institute of Neurological Sciences, Hospital Bellaria, Bologna, Italy; ^4^Department of Neurosciences and Rehabilitation, S. Anna University Hospital, Ferrara, Italy; ^5^Department of Biomedical & Specialty Surgical Sciences and Centre Haemostasis & Thrombosis, Section of Medical Biochemistry, Molecular Biology & Genetics, University of Ferrara, Ferrara, Italy; ^6^Department of Morphology, Surgery and Experimental Medicine, Vascular Diseases Center, University of Ferrara, Ferrara, Italy

**Keywords:** multiple sclerosis, whole exome sequencing, low-frequency variants, rare variants, *C6orf10*

## Abstract

In light of the complex nature of multiple sclerosis (MS) and the recently estimated contribution of low-frequency variants into disease, decoding its genetic risk components requires novel variant prioritization strategies. We selected, by reviewing MS Genome Wide Association Studies (GWAS), 107 candidate loci marked by intragenic single nucleotide polymorphisms (SNPs) with a remarkable association (*p*-value ≤ 5 × 10^-6^). A whole exome sequencing (WES)-based pilot study of SNPs with minor allele frequency (MAF) ≤ 0.04, conducted in three Italian families, revealed 15 exonic low-frequency SNPs with affected parent-child transmission. These variants were detected in 65/120 Italian unrelated MS patients, also in combination (22 patients). Compared with databases (controls gnomAD, dbSNP150, ExAC, Tuscany-1000 Genome), the allelic frequencies of *C6orf10* rs16870005 and *IL2RA* rs12722600 were significantly higher (i.e., controls gnomAD, *p* = 9.89 × 10^-7^ and *p* < 1 × 10^-20^). *TET2* rs61744960 and *TRAF3* rs138943371 frequencies were also significantly higher, except in Tuscany-1000 Genome. Interestingly, the association of *C6orf10* rs16870005 (Ala431Thr) with MS did not depend on its linkage disequilibrium with the *HLA-DRB1* locus. Sequencing in the MS cohort of the *C6orf10* 3′ region revealed 14 rare mutations (10 not previously reported). Four variants were null, and significantly more frequent than in the databases. Further, the *C6orf10* rare variants were observed in combinations, both intra-locus and with other low-frequency SNPs. The *C6orf10* Ser389Xfr was found homozygous in a patient with early onset of the MS. Taking into account the potentially functional impact of the identified exonic variants, their expression in combination at the protein level could provide functional insights in the heterogeneous pathogenetic mechanisms contributing to MS.

## Introduction

Multiple Sclerosis (MS) is a chronic autoimmune disease of the central nervous system (CNS), involving inflammatory-based mechanisms and characterized by demyelination, neurodegeneration and progressive accumulation of neurological dysfunction (Ciccarelli et al., 2014). The heterogeneous manifestation and clinical course of MS are explained by its complex multi-factorial nature, where the interaction of genetic, lifestyle, and environmental factors confer the susceptibility (Morandi et al., 2015; Olsson et al., 2017). The heritable contribution to MS risk is supported by investigations on families (Patsopoulos, 2018).

To date, the majority of genetic studies on MS have been focused on susceptibility variants. In particular, several genome-wide association studies (GWAS), and subsequent replication studies, have identified hundreds of variants within susceptibility gene loci (Bashinskaya et al., 2015).

The single nucleotide polymorphisms (SNPs) identified through GWAS are mainly located within non-coding regions of the genome, which could pinpoint the presence of disease-associated variants in linkage disequilibrium.

The very recent study, made by the International Multiple Sclerosis Genetics Consortium, provides for the first time the evidence that low-frequency variants (minor allele frequency, MAF < 5%) explain 11.34% of the observed difference between cases and controls (International Multiple Sclerosis Genetics Consortium, 2018). The majority of low-frequency variants which contributed to MS risk were not individually detectable at genome-wide thresholds and among those associated with MS, only 1/3 were in linkage disequilibrium with the common variants from the GWAS (International Multiple Sclerosis Genetics Consortium, 2018).

Genetic studies in MS have used whole exome sequencing (WES) to investigate somatic mutations (Kemppinen et al., 2014), to define the genetic contribution to MS clinical outcomes (Sadovnick et al., 2017) and to suggest new potential causative variants in families (Dyment et al., 2012; Garcia-Rosa et al., 2017; Maver et al., 2017; Mescheriakova et al., 2018) or unrelated patients (Bernales et al., 2018). WES data in families, suggesting monogenic disease forms caused by rare variants with strong functional impact (Ramagopalan et al., 2011; Wang et al., 2016), were not confirmed in the subsequent replication studies (Ban et al., 2013; International Multiple Sclerosis Genetics Consortium, 2016; Minikel and MacArthur, 2016). Based on the aforementioned findings, decoding the genetic risk components of MS still represents a challenge, and novel strategies are required to prioritize variants (Wang and Biernacka, 2015).

We set up a targeted WES-based pilot study in MS families, followed by low-frequency variants investigation in a cohort of unrelated patients in Italy, where a high prevalence and incidence of MS have been reported (Battaglia and Bezzini, 2017; Granieri et al., 2018).

The main aim of this study was to identify new exonic and potentially functional low-frequency variants for MS risk within genes marked by intragenic common variants from the GWAS.

## Materials and Methods

### Study Population

**FIGURE 1:**
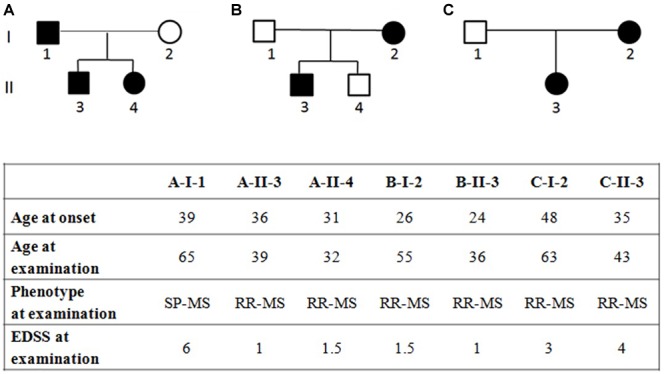
Pedigree of families **(A–C)** with multiple sclerosis and clinical characteristics of the affected family members, RR-MS, relapsing-remitting multiple sclerosis; SP-MS, secondary-progressive multiple sclerosis; PP-MS, primary progressive multiple sclerosis; EDSS, expanded disability status scale. EDSS ranges from 0 to 10 in 0.5-point increments; higher scores indicate more disability.

The study population included (i) three Italian families with MS-affected members (clinical characteristics and pedigree shown in Figure 1) and (ii) 120 Italian unrelated MS patients coming from the mainland, selected from previous studies (Gemmati et al., 2012; Marchetti et al., 2018; Ziliotto et al., 2018) for having age of MS onset under 52 and mean age of MS onset (34.5 ± 9.7) similar to that of the affected family members (34.1 ± 8.2; Student’s *t*-test *p*-value = 0.371). MS diagnosis was assessed according to the 2010 revised McDonald criteria (Polman et al., 2011). All MS patients underwent neurological visits, MRI examinations and assessment of the Expanded Disability Status Scale (EDSS) (Kurtzke, 1983). All patients were over 18 years of age and the patients’ medical records were examined in order to ascertain age of diagnosis and type of MS at the time of blood sample collection. Among the 120 unrelated MS patients, 44 patients were relapsing remitting MS (RR-MS), 53 were secondary progressive MS (SP-MS) and 23 had primary progressive (PP-MS) course of the disease. Written informed consent was obtained from all subjects, and the study was approved by the Ethical Committee of the S. Anna University-Hospital, Ferrara, Italy. Demographic and clinical characteristics of the unrelated MS patients are summarized in Table 1.

**Table 1:** Demographic and clinical characteristics of the unrelated MS patients cohort.

		Clinical phenotype			
	All MS	RR-MS	SP-MS	PP-MS	*p*-value
Sample size, n	120	44	53	23	
Female, n (%)	74 (61.7)	27 (61.4)	31 (58.5)	16 (69.6)	
Onset, mean y ± SD	34.5 ± 9.7	33.9 ± 9.3	32.9 ± 9.5	39.1 ± 9.8	0.032
EDSS at examination, median (IQR)	6 (2–6.5)	2 (1–2.5)	6.5 (6–6.5)	6 (6–6.5)	<0.0001

*RR-MS, relapsing-remitting multiple sclerosis; SP-MS, secondary-progressive multiple sclerosis; PP-MS, primary progressive multiple sclerosis; n, number; SD, standard deviation; y, years; EDSS, expanded disability status scale; IQR, inter quartile range. Descriptive analysis between RR-MS, SP-MS, and PP-MS was performed using ANOVA for age of onset, and Kruskal–Wallis test for EDSS at examination. Multiple comparison tests provided differences in age of onset between SP-MS and PP-MS (*p* = 0.031, Bonferroni’s test), and in EDSS of RR-MS with SP-MS and with PP-MS (both *p* < 0.0001, Dunn’s test).*

### Search Strategy and Selection Criteria of Candidate Genes

**FIGURE 2:**
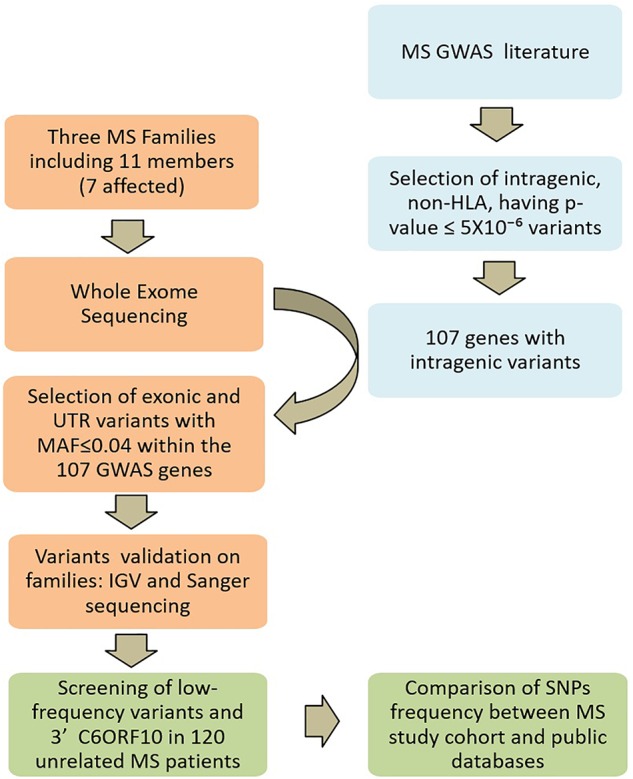
Study design. Multiple sclerosis (MS) GWAS and WES SNP filtering is schematically described. In light blue, the search strategy and selection criteria of candidate genes; in orange the Whole-exome sequencing and the analysis on MS families; in green the screening strategy in the unrelated MS patients.

A systematic review of the literature was performed for all years available through December 31, 2017. The primary source was the PubMed database^1^, for which search terms “GWAS” and “multiple sclerosis” were used. Further search included NHGRI-EBI GWAS catalog^2^. Variants identified by GWAS in MS were selected for being intragenic, non-HLA, and having *p*-value ≤ 5 × 10^-6^. On the basis of these selected common variants, we generated the final gene reference list for the present study (Supplementary Table 1). The study design is schematically described in Figure 2.

### Whole-Exome Sequencing and Analysis

The genomic DNA (gDNA) was extracted from peripheral blood using the Wizard^®^ Genomic DNA Purification Kit (Promega, Madison, WI, United States). WES was performed on eleven individuals, seven diagnosed with MS and four unaffected, from three independent families (Figure 1). Sequencing was performed by BGI (Shenzhen, China) using nanoarray-based short-read sequencing-by-ligation technology (cPAL^TM^). Reads were mapped against the hg19 human reference sequence^3^ using SOAPaligner^4^. Variants calling was performed with the Complete Genomics Small Variant Caller^5^. Genetic variations were verified in: (i) the database of Single-Nucleotide Polymorphisms (dbSNP, Build 150^6^), (ii) the 1000 Genomes Project databases^7^, (iii) the Exome Aggregation Consortium (ExAC^8^), and (iv) controls of Genome Aggregation database (gnomAD v2.1.1 controls^9^).

The coverage of the target region was 99.26%, the average depth on target region was 129X and the target coverage with at least 20× was 90.6%. The filtering performed on WES-data is schematically described in Figure 2. In order to remove systematic artifacts, we visually verified the filtered low-frequency variants with IGV^10^. We considered a variant as true heterozygous with a call of 40% of the total reads.

The effects of new coding variations on protein structure and function were predicted using Provean/SIFT^11^, while the effects of low-frequency coding variations, already reported on databases as prediction by SIFT/PolyPhen, were extrapolated from Ensembl^12^. The whole-exome sequencing dataset generated during the current study is available in the https://www.ncbi.nlm.nih.gov/sra (Accession Number: PRJNA544162).

### Mutation Screening

Low-frequency variants, identified through the filtering in MS families, were confirmed by Sanger sequencing (*n* = 13) or restriction analysis (*n* = 2, *ADAMTS3* and *GC*). Primers to amplify the coding sequences containing the identified low-frequency variants were designed with Primer3 software v0.04.0^13^. A total of 50 ng of gDNA was amplified by polymerase chain reaction (PCR) using a standard protocol with AmpliTaq Gold 360 DNA polymerase (Applied Biosystems, Foster City, CA, United States) (Balestra et al., 2016). PCR conditions were set up as follows: an initial denaturation at 94°C for 5 minutes and then at 65°C for 3 minutes, followed by 35 cycles at 94°C for 30 seconds, specific temperatures for each couple of primers for 30 seconds, 72°C for 30 second or 1 minute, and a final elongation at 72°C for 7 minutes. Detailed primer sequences and PCR conditions used for Sanger sequencing are reported in Table 2. The PCR products were purified with CleanSweep^TM^ PCR Purification (Applied Biosystems) prior to direct sequencing (Macrogen, Madrid, Spain). Sequences were analyzed using the software NovoSNP (Weckx et al., 2005). Size of PCR amplicons and restriction products were examined through agarose gel electrophoresis.

**Table 2:** Primer sequences and PCR conditions used for Sanger sequencing.

Gene	SNP position GRCh37/hg19	SNP ID	Forward primer sequence (5′-3′)	Reverse primer sequence (5′-3′)	Tm (°C)	Length (bp)
*ANKRD55*	5:55407449	rs77017041	TTGTCACTCCAGTTCCTAGCTT	CCTGATGAAGCATGTGGAAT	60	850
*C6orf10*	6:32261153	rs16870005	TTTAGGCAATGGCTGGGATA	TGTGCCAAGAAGACAGGAATC	60	658
*CD86*	3:121774281	rs11575853	TCTTCCTCAAGTGTGGTCAAAA	GCACCATCTTCAACCTCAGC	60	297
*EVI5*	1:92979432	rs41286809	TGGCAATGGTAAATCAGTGG	CATGGAATGTTTGCTTTTTGG	60	595
*IL2RA*	10:6054765	rs12722600	ATAGAGACAAGGTTGCCACTGC	CCACAGCTATTGTCTGCCATATAAA	66	468
*MALT1*	18:56367823	rs74847855	CACTTTCAAAGCTTCATACTGAAATC	AAGACAAAACACATGGATCAAATCT	60	427
*MMEL1*	1:2530169	rs147248515	TAACCCCTCATGTCCCACAC	GGGGCTGGGTTTCTTAGATT	63	353
*STAT4*	2:191899319	–	GAAATTCTCAAAACCCCATGT	AAATTGAGCACAAAATTGAAGC	60	209
*TET2*	4:106156163	rs61744960	TATTATCCAGATTGTGTTTCCATTG	CTTAGTGAACACTGAGCTTTGCTT	63	471
*TOP3A*	17:18217958	rs2230153	TCGCCTTCATCTCGATTCTT	TGAGCCTCATCTCTGGCTTC	55	342
*TRAF3*	14:103371923	rs138943371	ATGTGTGCCAGGGTCTACCT	TCTTGAAGCTGCTGCTGTTG	63	220
*WWOX*	16:78458807	rs7201683	AAAGAATTTCTCATTCCCGAAG	CACCCACATGTCTCAAGCAG	60	444

The presence of the selected low-frequency variants was investigated in a sample set of 120 Italian unrelated MS patients through Sanger sequencing (Table 2) or restriction analysis using the primer sequences and the restriction enzymes reported in Table 3.

**Table 3:** Primer sequences and restriction enzymes used for restriction analysis.

Gene	SNP position GRCh37/hg19	SNP ID	Forward primer sequence (5′-3′)	Reverse primer sequence (5′-3′)	Tm (°C)	Ref allele SNP allele	Restriction enzyme
*ADAMTS3*	4:73414590	–	TCACCCCACAGATTTACCA**T**TA	GGGCTTTAGTCGCAGATGAA	60	A:139+46+20 G:159+46	*MseI*
*ANKRD55*	5:55407449	rs77017041	GGTGATGATGTCATTGACT**G**CTG	TACTCACATATCATCCCTGCTCTTT	60	A:201+21 G:222	*Bpu10I*
*CD86*	3:121774281	rs11575853	CTGCTGTAACAGGGACTAGC**T**CA	AGGAACTAAGTGAAGGACACACATC	60	A:176+23 G:199	*Hpy188I*
*EVI5*	1:92979432	rs41286809	ACACATAGAAGGCACTCAAAAATTAG	CTATAAAATCTTCATCGGAGGA**C**TG	60	C:250+25 T: 275	*Bsr I*
*GC*	4:72669661	rs76781122	CCACTAATGCCAGCCAATCT	TGCTTTGCACAGAAATCCTC	60	G:361+48 T:266+95+48	*ApoI*
*IL2RA*	10:6054765	rs12722600	AACAGAAGTCATGAAGCCCA**C**GT	AGTGGTTTTGCCCTTCCTC	60	G:219+21 A:240	*PmlI*
*MALT1*	18:56367823	rs74847855	CACTTTCAAAGCTTCATACTGAAATC	AAGACAAAACACATGGATCAAATCT	60	A:248+179 G:427	*Hpy188III*
*MMEL1*	1:2530169	rs147248515	CACTAAAGCTTAACCCCTCATGTC	TATCCTCTGTCAAAATCAAGCTG**G**T	60	G:221+27 T:248	*BanI*
*TET2*	4:106156163	rs61744960	CTGATGATGCTGATAATGCCAGT	GTAAGCACCATTCATTTCATTTTGT	60	G:134+75+39 A:134+114	*NlaIV*
*TOP3A*	17:18217958	rs2230153	TCGCCTTCATCTCGATTCTT	TGAGCCTCATCTCTGGCTTC	55	G:190+134+18 A:190+152	*EaeI*
*TRAF3*	14:103371923	rs138943371	ATGTGTGCCAGGGTCTACCT	TCTTGAAGCTGCTGCTGTTG	63	C:146+62 T:208	*AvaII*
*TYK2*	19:10472452	rs12720355	GGACCCTAGTCACCATGA**G**AT	GTCTCGTAGAAGGCCTGTGG	60	C:197+18 T:215	*MboI*
*WWOX*	16:78458807	rs7201683	AAAGAATTTCTCATTCCCGAAG	CACCCACATGTCTCAAGCAG	60	C:444 G:259+185	*RsaI*

*To verify the presence of the selected mutations, PCR products were digested with restriction enzymes. Nucleotides in bold and underlined are those modified to create specific restriction sites.*

### Bio-Informatics Analysis of Nucleotide Changes

The prediction of mi-RNA targets was conducted by using the tool at www.mirdb.org exploiting the support vector machines (SVMs) procedures. The computational prediction of splice sites and or splicing regulatory elements was conducted by using the www.umd.be/HSF/ online software.

### Statistical Analysis

For populations comparison, we used MAFs obtained from: (i) the “dbSNP Build 150” (Homo sapiens Annotation Release 108) which combines all available frequencies from submitted SNPs clustered together into a reference SNP, (ii) the ExAC, which includes exome sequencing data from a wide variety of large-scale sequencing projects and in particular of European (not-Finnish) individuals, (iii) controls of gnomAD, which includes only samples from individuals who were not selected as a case in a case/control study of common disease, and (iv) 1000 Genome Project which contains allelic frequencies for a sample of 107 subjects from Tuscany, Italy, an optimal reference population for our MS individuals. The low prevalence (188 per 100’000 individuals) of MS in Tuscany (Bezzini et al., 2016) makes improbable the presence of individuals with MS in the Tuscany control sample.

To test the difference in MAFs between reference populations and the allelic frequencies observed in the study population, a two-proportion *z*-test, with a 0.05 two-sided significance level, was applied. A threshold of *p* < 0.0042, assuming the Bonferroni correction for multiple testing, was used for significance.

The potential enrichment of exonic low-frequency variants in MS patients was evaluated using a permutation approach based on the observed exonic polymorphisms. We first generated the null distribution of the number of low-frequency variants in a random sample of 107 genes, considering the exons composing the longest isoform of each gene, as defined by the human genome annotation (GRCh37/hg19). We took into account both the number and the length of exons, dividing the number of low-frequency variants by the total exon length, for each gene set. Then, we repeated the permutation process 1,000 times and the empirical *p*-value was defined as the proportion of replicates showing a number of variants higher than the observed value.

## Results

### Selection of Candidate Genes

Since GWAS and classical linkage studies have extensively investigated the *HLA* locus, harboring the greatest genetic risk for MS (reviewed in Hollenbach and Oksenberg, 2015), *HLA* genes were not included in this study.

The review of GWAS in MS literature, reporting polymorphisms associated with MS in case-control studies, identified 141 variants which were selected for being intragenic and for having a *p*-value ≤ 5 × 10^-6^, an arbitrary threshold potentially highlighting genes with remarkable disease association. These common variants established the list of 107 genes used for the purpose of this study. Variants and corresponding genes are listed in the Supplementary Table 1.

### Search for Low-Frequency Variants in MS Families by WES

WES was performed in three independent Italian families with at least two affected members in each pedigree (Figure 1).

A targeted analysis within the 107 MS susceptibility genes was conducted in all pedigree members. SNPs with MAF ≤ 0.04 were taken into account when present in at least one affected family member. The selection of SNPs with MAF ≤ 0.04 was aimed at filtering low-frequency variants which did not emerge in GWAS.

These filtering criteria revealed 17 exonic mutations (ten missense and seven synonymous) and three in the UnTranslated Regions (UTRs), all in the heterozygous condition (Table 4).

**Table 4:** List of low-frequency variants identified in the MS families.

Gene	SNP position GRCh37/hg19	SNP ID	MAF % dbSNP150	MAF % ExAC European (not Finnish)	MAF % controls GnomAD European (not Finnish)	Mutation type	SNPs carriers
*ADAMTS3*	4:73178175	rs150270324	0.926	1.34	1.12	Missense	A-I-2+ A-II-3
*ADAMTS3*	4:73414590	–		0	II-3
*ADAMTS3*	4:73414590	–		0	0	Missense	B-I-1+ B-II-3+ B-II-4
***ANKRD55***	5:55407449	rs77017041	0.427	0.63	0.71	Missense	C-I-2+C-II-3
*BTNL2*	6:32363893	rs28362679	1.825	–	0	Missense	B-I-1+ C-I-1+ C-I-2
***C6orf10***	6:32261153	rs16870005	1.250	1.43	0.85	Missense	B-I-2+ B-II-3
***GC***	4:72669661	rs76781122	1.611	3.35	2.77	Missense	B-I-2+ B-II-3
*MALT1*	18:56367823	rs74847855	3.734	4.28	4.20	Missense	B-I-2+ B-II-3+ B-II-4
***MMEL1***	1:2530169	rs147248515	0.022	0.028	0.037	Missense	B-I-2+ B-II-3
***TET2***	4:106156163	rs61744960	2.641	3.77	3.69	Missense	B-I-2+ B-II-3+ C-I-2+C-II-3+ A-I-1+A-II-4
*WWOX*	16:78458807	rs7201683	1.989	1.23	1.11	Missense	B-I-2+ B-II-3+ B-II-4+ C-I-2
***EVI5***	1:92979432	rs41286809	1.116	1.55	1.61	Synonymous	C-I-2+C-II-3+ A-I-1+ A-II-3+ A-II-4
*GC*	4:72620788	rs76803094	1.799	2.56	2.14	Synonymous	B-I-1+ B-II-3+ B-II-4
*GEMIN2*	14:39587220	rs150986614	0.251	0.37	0.37	Synonymous	A-I-2+ A-II-3+ A-II-4
***STAT4***	2:191899319	–		–	–	Synonymous	C-I-2+C-II-3
***TRAF3***	14:103371923	rs138943371	0.245	0.30	0.36	Synonymous	C-I-2+C-II-3
*TOP3A*	17:18217958	rs2230153	1.692	0.46	0.36	Synonymous	C-I-2+C-II-3+ A-I-2
***TYK2***	19:10472452	rs12720355	0.962	1.38	1.45	Synonymous	C-I-2+C-II-3
*CD86*	3:121774281	rs11575853	1.078	–	3.1	UTR 5′	B-I-2+ B-II-3+ C-I-1
*IL2RA*	10:6054765	rs12722600	1.777	–	0.87	UTR 3′	B-I-2+ B-II-3+ C-I-1+ C-II-3
*RRAS2*	11:14300827	–		–	–	UTR 3′	B-I-2+ B-II-4

*In bold the genes including the exonic variants present only in affected family members defined as in Figure 1.*

Among the 20 exonic and UTRs variants, nine were present only in the affected members of the families (rs77017041, *ANKRD55*; rs16870005, *C6orf10*; rs41286809, *EVI5*; rs76781122, *GC*; rs147248515, *MMEL1*; the new synonymous variant on *STAT4*; rs61744960, *TET2*; rs138943371, *TRAF3*; rs12720355, *TYK2*) and 14 variants were detected in at least two affected family members with parent-child transmission.

The number of low-frequency exonic variants in these families was investigated by a permutation test. No significant difference was observed between our result and that expected by chance (*p* = 0.231).

### Screening of WES-Selected Low Frequency Variants in Unrelated Multiple Sclerosis Patients

We focused our investigation on the 14 low-frequency variants with parent-child transmission and on the new variant of *ADAMTS3* (Table 5). The 15 candidate SNPs were explored in a sample set of 120 Italian unrelated MS patients (Table 1).

**Table 5:** Selected rare variants in the cohort of 120 unrelated multiple sclerosis patients.

Gene	SNP position GRCh37/hg19	SNP ID	Transcript (exon)	Amino acid change	SIFT/ PolyPhen prediction	Nucleotide change	MAF % in MS patients alleles *n* = 240 (n alleles)	MAF % 1000 GP (Tuscany) alleles *n* = 214	MAF % dbSNP150	MAF % ExAC European (not Finnish)	MAF % controls GnomAD European (not Finnish)	*P*-value° (Tuscany)	*P*-value° (dbSNP150)	*P*-value°(ExAC)	*P*-value° (controls GnomAD)
*ADAMTS3*	4:73414590	–	ENST0000 0286657 (3/22)	Lys37Glu	0.17/-0.01^$^	T > C	0(0)	–	–	–	–	–	–	–	–
*ANKRD55*	5:55407449	rs77017041	ENST0000 0341048 (10/12)	Ser376Pro	0.01/0.767	A > G	0(0)	0.47	0.427	0.63	0.71	0.2871	0.3103	0.2174	0.1902
*C6orf10*	6:32261153	rs16870005	ENST000 00533191 (26/26)	Ala431Thr	0.46/0.028	C > T	3.75 (9)	1.4	1.250	1.43	0.85	**0.0019**	**0.00049**	**0.0025**	**9.89 × 10**^-^**^07^**
*CD86*	3:121774281	rs11575853	ENST000 00330540 (5′UTR)	–56bp from +1 Met	n.a.	A > G	3.75 (9)	2.8	1.078	–	3.1	0.3723	**6.1 × 10**^-^**^5^**	–	0.5612
*EVI5*	1:92979432	rs41286809	ENST000 00540033 (18/18)	Phe749Phe	–	G > A	0.83 (2)	0.47	1.116	1.55	1.61	0.4105	0.6768	0.3688	0.3391
*GC*	4:72669661	rs76781122	ENST0000 0504199 (1/14)	Met1Ile	0.2/0 Start codon	G > T	4.58 (11)	5.61	1.611	3.35	2.77	0.4895	**0.00025**	0.2883	0.0869
*IL2RA*	10:6054765	rs12722600	ENST000 00379959 (3′UTR)	+70 from 273 stop	n.a.	G > A	7.92 (19)	4.21	1.777	–	0.87	**0.0042**	**6.0 × 10**^-^**^13^**	–	**<1.0 × 10**^-^**^20^**
*MALT1*	18:56367823	rs74847855	ENST000 00348428 (4/17)	Arg217Gly	0.69/0	A > G	4.58 (11)	1.9	3.734	4.28	4.20	**0.0019**	0.4877	0.8164	0.7672
*MMEL1*	1:2530169	rs147248515	ENST000 00378412 (12/24)	Pro368Thr	0.14/0.326	G > T	0(0)	0	0.022	0.028	0.037	–	–	–	–
*STAT4*	2:191899319	–	ENST000 00392320 (18/24)	Gln525Gln	–	A > G	0^∗^ (0)	–	–	–	–	–	–	–	–
*TET2*	4:106156163	rs61744960	ENST000 00540549 (3/11)	Gly355Asp	0.01/0.282	G > A	8.33 (20)	5.6	2.614	3.77	3.69	0.0667	**2.8 × 10**^-^**^08^**	**0.00021**	**0.00014**
*TOP3A*	17:18217958	rs2230153	ENST000 00542570 (1/19)	Ala45Ala	–	G > A	1.66 (4)	0.47	1.692	0.46	0.36	**0.0067**	0.9757	**0.0057**	**0.00073**
*TRAF3*	14:103371923	rs138943371	ENST000 00347662 (11/11)	Ser478Ser	–	C > T	1.25 (3)	0.47	0.245	0.30	0.36	0.0773	**0.00164**	**0.0071**	**0.02133**
*TYK2*	19:10472452	rs12720355	ENST00 000525621 (13/25)	Ile651Ile	–	C > T	1.66 (4)	1.4	0.962	1.38	1.45	0.7251	0.2634	0.7034	0.77887
*WWOX*	16:78458807	rs7201683	ENST000 00566780 (7/9)	Leu216Val	0.19/0.04	C > G	2.08 (5)	0.47	1.989	1.23	1.11	**0.00026**	0.9166	0.2304	0.150086

*The SIFT and PolyPhen scores (0.0 to 1.0) have opposite meanings. Prediction of the SIFT score ranges 0.0 to 0.05 deleterious; 0.05 to 1.0 tolerated. Prediction of the PolyPhen score ranges: 0.0 to 0.15, benign; 0.15 to 1.0 possibly damaging; 0.85 to 1.0 damaging. n.a., not applicable. ^*$*^SIFT/Provean prediction for *ADAMTS3* = (0.17) tolerated/(-0.01) neutral. ^∗^Investigated in 218 alleles. °Bonferroni’s correction *p*-value set to 0.0042. Significant *p*-values are in bold.*

The predicted effects of the 15 analyzed low-frequency variants, each on the main encoded transcript, are reported in Table 5. In addition to the mutation of the start codon (*GC*, Met1Ile), expected to reduce the amount of the translated protein, missense variants were predicted as damaging (*ANKRD55* Ser376Pro; *TET2* Gly355Asp) or potentially damaging (*MMEL1* Pro368Thr). For the rs147248515 (*MMEL1*) the proline to threonine change produced discrepant predictions. Among the four SNPs predicted as benign, two cause noticeable changes in amino acid polarity and size (Arg to Gly in *MALT1*; Ala to Thr in *C6orf10*).

Aimed at prioritizing low-frequency variants that may contribute to the disease-risk, we compared the MAFs observed in the unrelated MS cohort with those reported in public databases (Table 5). The new variants on *STAT4* and *ADAMTS3* genes were not found in the cohort of unrelated MS. The allelic frequencies of *C6orf10* rs16870005 and *IL2RA* rs12722600 resulted significantly higher in MS patients compared with all the databases (even after Bonferroni’s correction). Based on frequencies in the Control gnomAD, the OR for the rs1687005 risk T-allele was 4.57 (95% CI 2.33–8.97) and the odds ratios for the rs12722600 risk T-allele was 9.88 (95% CI 5.71–17.09), both highly significant (*p*-value < 0.001).

The *TET2* rs61744960 and *TRAF3* rs76781122 showed significant MAF differences between MS patients and public databases with the exception of a nominal borderline *p*-values with the Italian Tuscany population. On the other hand, *TOP3A* rs2230153 showed significant MAF differences between MS patients and public databases, with the exception of dbSNP150.

For two SNPs (*CD86*, rs11575853 and *GC*, rs76781122) significant differences were observed only in the comparison between MS patients and the dbSNP150 population. Of note, for the *MALT1* rs74847855, and *WWOX* rs7201683 highly significant MAFs differences between MS and Tuscany subjects were observed, that may reflect increased frequency of low-frequency alleles in MS Italian patients.

Within the unrelated MS cohort, 17 patients were carriers of two low-frequency variants and five patients were carrier of three variants. The combinations repeatedly included the *C6orf10, TET2*, and *IL2RA* variants (Table 6). The variants detected in combination were always located on different chromosomes.

**Table 6:** Unrelated multiple sclerosis patients carriers of at least 2 low-frequency variants.

ID	Gender	Age of MS onset	Phenotype at examination	*C6orf10* rs16870005	*CD86* rs11575853	*EVI5* rs41286809	*GC* rs76781122	*IL2RA* rs12722600	*MALT1* rs74847855	*TET2* rs61744960	*TOP3A* rs2230153	*TRAF3* rs138943371	*TYK2* rs12720355	*WWOX* rs7201683
132 ZM	F	20	RR							Het	Het			
159 ZM	M	26	RR							Het	Het			
173 ZM	F	31	RR							Het	Het			
**194 ZM**	**F**	**31**	**RR**				Het	Hom^∗^						Het
27-WP3	M	31	SP	Het			Het							
43-WP3	F	31	PP		Het			Het						
155 ZM	F	33	SP							Het		Het		
51-WP3	M	33	SP	Het						Het				
109 ZM	F	37	SP		Het	Het								
**63-WP3**	**F**	**37**	**SP**	Het	Het					Het				
**115 ZM**	**F**	**38**	**SP**						Het	Het				Het
69-WP3	M	40	SP				Het		Het					
**111 ZM**	**F**	**41**	**RR**	Het				Het		Het				
49-WP3	F	42	PP				Het	Het						
**57-WP3**	**M**	**42**	**SP**	Het				Het					Het	
208 ZM	F	44	RR					Het						Het
MS18	F	45	SP		Het							Hom^∗^		
184 ZM	M	47	SP					Het						Het
192 ZM	F	48	RR	Het				Het						
72-WP3	F	48	PP						Het	Het				
204 ZM	M	50	RR					Het	Het					
MS07	M	n.d.^§^	SP		Het						Het			

*The heterozygous (Het) or homozygous (Hom^∗^) condition of the variants is specified. In bold, patients with combination of 3 low-frequency variants. ^*§*^Age of onset below 50 years old.*

The *IL2RA* rs12722600 and the *TRAF3* rs138943371 were detected in the homozygous condition.

The comparison of age of MS onset between patients with or without the 15 investigated low-frequency variants did not provide significant differences.

### Detection of Null Mutations in the 3′ Exon of *C6orf10*

**FIGURE 3:**
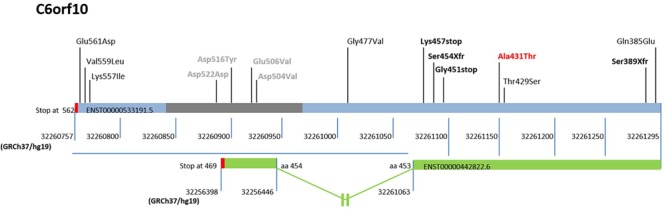
Schematic representation of the sequenced region and mutations in the 3′ exon of *C6orf10*. The low-frequency variants discovered by 3′ exon sequencing in the unrelated MS are reported upper the bar (light blue/gray) of the ENST00000533191.5 transcript, used as reference. The gray bar indicates the repetitive regions, and the red bars the translational stop codons of transcripts. The reference nucleotide position in GRCh37/hg19 is also shown below the transcript bars. The first variant identified by our WES is reported bold and red. The stop and frame shift variants are highlighted in bold and black. The transcript ENST0000442822.6, the only one undergoing splicing and ending at position 32256398 of Chr 6, is represented as a green bar. A blue horizontal line highlights the 3′ region with the different reference nucleotide position for each transcript. The out of scale intron is indicated with an interruption symbol. The different numbering of this transcript in the 3′ region is reported. The change in the reference nucleotide position in the ENST0000442822.6 starts after the aminoacid (aa) 453 at position 32261063.

The rs16870005 within *C6orf10*, a scarcely investigated locus, resulted the only missense variant with a significantly increased frequency in our cohort compared to dbSNP-Build150, Tuscany of 1000 Genome Project, ExAC -European (not Finnish)- and Control gnomAD -European (not Finnish)- (Table 5) and, in addition, it was frequently present in combination with other low-frequency variants (Table 6). Further, the nucleotide change C > T (rs16870005) in the 3′ region of the *C6orf10* transcripts would substitute threonine for alanine in the carboxyl-terminal region of all the predicted proteins (reference transcript used for the study shown in Figure 3).

Based on these observations we sequenced the region chr6:32261295-32260757 in the 120 MS patients, which revealed the presence of 14 low-frequency mutations (MAF ≤ 0.04), 10 not previously reported (Table 7).

**Table 7:** Low-frequency variants in 3′ exon of *C6orf10* detected by Sanger sequencing within the cohort of 120 unrelated MS patients.

SNP position GRCh37/hg19	SNP ID	Amino acid change	Provean/SIFT prediction	Nucleotide change	MAF % dbSNP150	MAF % ExAC European (not Finnish)	MAF % Controls GnomAD European (not Finnish)	MAF % in 120 MS patients (n alleles)
6:32260761	–	Glu561Asp	(-1.16) neutral/ (0) damaging	C > G	–	–	–	0.42 (1)
6:32260769	–	Val559Leu	(-0.86) neutral/ (0.31) damaging	C > G	–	–	–	0.42 (1)
6:32260774	–	Lys557Ile	(-1.6) neutral/ (0.004) damaging	T > A	–	–	–	0.42 (1)
6:32260878^∗^	–	Asp522Asp	neutral/tolerated	G > A	–	–	–	1.25 (3)
6:32260898^∗^	–	Asp516Tyr	(-2.38) neutral/ (0.011) damaging	C > A	–	–	–	0.42 (1)
6:32260927^∗^	–	Glu506Val	(-1.42) neutral/ (0.028) damaging	T > A	–	–	–	0.42 (1)
6:32260933^∗^	rs766126891	Asp504Val	(-2.42) neutral/ (0) damaging	T > A	0.001	–	–	0.42 (1)
6:32261014	**rs7751028**	Gly477Val	(-3.43) deleterious/ (0.008) damaging	C > A	2.480	0.98	0.99	0.42 (1)
6:32261075	–	Lys457stop	Damaging	T > A	–	–	–	0.42 (1)
6:32261084	–	Ser454Xfr	Damaging	A > insG	–	–	–	0.42 (1)
6:32261093	–	Gly451stop	Damaging	C > A	–	–	–	0.42 (1)
6:32261158	rs114543649	Thr429Ser	(1.30) neutral/ (1) tolerated	G > C	2.479	0.98	0.99	0.42 (1)
6:32261277	–	**Ser389Xfr**	Damaging	T > delT	–	–	–	0.83 (2)
6:32261291	–	Gln385Glu	(1.38) neutral/ (1) tolerated	G > C	2.482	0.98	0.99	0.42 (1)

*The sequenced 3′ exonic region spans chr6:32261295-32260757 (GRCh37/hg19). The reference transcript is ENST00000533191.5, and the reference protein is ENSP00000431199 (exon position 26/26). The Provean cut off: equal or below -2.5, deleterious; above -2.5, neutral. The SIFT score range prediction: 0.0 to 0.05 deleterious; 0.05 to 1.0 tolerated. All variants were detected in heterozygous condition with the exception of Ser389Xfr (homozygous condition, bold and black). ^∗^Position in repetitive regions.*

Of note, two mutations predicted premature termination of translation and two translational frameshift. Inspection of *C6orf10* variants (Figure 3) pointed out that the four null mutations affected all C6orf10 transcripts. Among these, the ENST0000442822.6, after splicing, is shorter and encodes a different 3′ sequence. In the transcripts other than ENST0000442822.6, null mutations would remove a larger C-terminus portion (Figure 3) in which we detected several missense SNPs.

For missense changes the algorithms predicted discordant effects (Table 7), with the exception of the damaging Gly477Val (rs7751028).

Several databases were inspected for low-frequency (MAF ≤ 0.04) exonic variants within the full *C6orf10* transcript ENST00000533191.5 (total length 80 Kb), and for the presence of null variants. In particular, (i) in the Control gnomAD database 301 variants were found, of which 24 were nonsense or frameshift, and (ii) in the ExAC database 271 variants were found, of which 20 were nonsense or frameshift. The proportion of null variants in the MS patients (4/14 within 538 bp) was higher than that in the Control gnomAD (*p* = 0.0254, Maximum Likelihood chi-square) and in the ExAC (*p*-value = 0.0184, Maximum Likelihood chi-square).

The distribution of the 14 low-frequency *C6orf10* variants detected by Sanger sequencing in the unrelated MS patients is shown in Table 8. The frameshift mutations, of which the Ser389Xfr in the homozygous condition, were detected in two patients with young age of disease onset (21 and 25 years). Three patients were carriers of two/three missense *C6orf10* mutations (Table 8). None of the 14 low-frequency variants was associated with the presence of the *C6orf10* rs16870005.

**Table 8:** Low-frequency variant genotypes in 3′ exon of *C6orf10* within the unrelated MS patients.

ID	Gender	Age of MS onset	Phenotype at examination	*C6orf10*
138 ZM	F	21	RR	**Ser454Xfr (Het)**
221 ZM	M	22	RR	Val559Leu (Het) Glu561Asp (Het)
150 ZM	F	25	RR	**Ser389Xfr (Hom)**
194 ZM	F	31	RR	Gln385Glu (Het) Thr429Ser (Het) Gly477Val (Het)
106 ZM	M	32	SP	Asp504Val (Het)^∗^ Asp522Asp (Het)^∗^
128 ZM	F	32	RR	Glu506Val (Het)^∗^
109 ZM	F	37	SP	Asp522Asp (Het)^∗^
115 ZM	F	38	SP	**Lys457stop (Het)**
MS23	F	38	RR	**Gly451stop (Het)**
112 ZM	F	41	RR	Lys557Ile (Het)
25-WP3	F	51	PP	Asp516Tyr (Het)^∗^
65-WP3	F	51	SP	Asp522Asp (Het)^∗^

*MS patients carrying low-frequency *C6orf10* variants (within the region chr6:32261295-32260757) are reported. The heterozygous (Het) or homozygous (Hom) condition of the variants is specified. In bold and black, the *C6orf10* stop and frame shift variants. ^∗^*C6orf10* variants in repetitive regions.*

Seven patients over the 11 carriers of the *C6orf10* variants were also carriers of SNPs detected in the family WES study (Supplementary Table 2).

## Discussion

Taking into account the complex multi-factorial nature of MS, and the recently estimated contribution of low-frequency variants into disease risk, we aimed at investigating genetic risk components through combination of variant prioritization strategies. We explored by WES 107 candidate loci for exonic low-frequency variants in three Italian MS families with two or three affected members and validated the results in 120 unrelated Italian patients. This experimental approach brought to attention 15 exonic variants, firstly selected for parent-child transmission and further investigated for increased frequency as compared to public databases. Among these, *STAT4* and *ADAMTS3* variants were found to be private of the families under study, which might support the notion that a proportion of the unexplained MS heritability is accounted by additive effects of individual variants (International Multiple Sclerosis Genetics Consortium, 2018).

Noticeably, our screening identified a number of significant differences in the observed allelic frequencies as compared with public databases. The observed MAFs, higher in the Italian MS patients under study, point toward an increase in frequency of the detected variants in patients as compared to healthy population. The *C6orf10* rs16870005 and *IL2RA* rs12722600 are the main signals identified in our study, as confirmed by comparison with all databases.

Through different experimental approaches, focused on low-frequency variants, we report several and novel findings in the *C6orf10* locus. The 3′ region, including the potentially damaging variant rs16870005 which resulted associated with MS in our cohort, was found to contain 14 low-frequency mutations, among which 10 not previously reported and four potentially null variants. Furthermore, three patients showed combination of the *C6orf10* heterozygous low-frequency variants, one was found to be homozygous for the Ser389Xfr, and seven displayed combinations of *C6orf10* with low-frequency variants in other candidate genes. Although anecdotal, finding the homozygous Ser389Xfr in a patient with early onset of the MS disease fosters further investigation in relation to the recent study suggesting that *C6orf10* could be implicated in the age of onset of other neurodegenerative disease (Zhang et al., 2018).

The interpretation of potential functional consequences was hampered by the *C6orf10* chromosomal location and structure. As a matter of fact, this ORF is located on chromosome 6p21.32, in the major histocompatibility complex region which contains the major MS-associated risk gene *HLA-DRB1* (Bashinskaya et al., 2015). To evaluate if the signals of associations between the *C6orf10* variants could reflect linkage disequilibrium (LD) with *DRB1* SNPs reported in the literature (reviewed in Bashinskaya et al., 2015), the data from the 1000 Genome project were explored (Supplementary Table 3). The low LD of *C6orf10* rs16870005 with *C6orf10* rs3129934 (r^2^ 0.113), and with the *HLA* rs9271366 (r^2^ 0.055), suggests that the association between *C6orf10* rs16870005 and MS does not simply reflect the LD with the *HLA-DRB1* locus.

The *C6orf10* structure comprises several transcripts, including three isoforms of a validated, but not characterized, long non-coding RNA (NR_136244.1, NR_136245.1, and NR_136246.1) and a pseudogene hnRNP (*HNRNPA1P2*). Thus, the null mutations that we have found in the MS cohort would affect the C-terminal portion of several uncharacterized proteins expressed in brain and B cells ^14^both tissues of interest for MS.

The small sample size and the statistical power derived from our population do not permit an informative evaluation of the possible impact of the numerous newly detected *C6orf10* variants on the disease onset or clinical course within an integrated multiple variants model.

Finding of the *IL2RA* 3′UTR low-frequency variant (rs12722600) (i) in two MS families, (ii) with a significantly higher frequency in the unrelated MS cohort than in the public databases, and (iii) in several combinations with the other low-frequency SNPs, is particularly intriguing. As for the other *IL2RA* polymorphisms previously associated with the risk of developing the disease (Matiello et al., 2011; Wang and Chen, 2018), the functional consequence of the rs12722600 can be only proposed (Maier et al., 2009) as affecting the post-transcriptional regulation of the *IL2RA* mRNA, of which little is known (Techasintana et al., 2017). Bio-informatics prediction of miRNA binding sites in the *IL2RA* 3′UTR did not reveal creation or disruption of regulatory sites produced by the rs12722600 nucleotide change. Interestingly, therapies for MS have been already developed to avoid the formation of the interleukin-2 receptor complex, and particularly targeting CD25 (Bielekova, 2018), the α-subunit encoded by the *IL2RA.* Our findings support further studies aimed at characterizing the *IL2RA* 3′UTR in patients undergoing this therapeutic approach.

The *TET2* gene codifies for an enzyme that catalyzes the conversion of 5-methylcytosine to 5-hydroxymethylcytosine, thus modifying the DNA methylation pattern. As functional partner, TET2 may participate in histone modification (O-GlcNAcylation, Chen et al., 2013). Noteworthy, demethylation by TET2 is also actively involved in T cells differentiation and their cytokines production (Ichiyama et al., 2015; Wang et al., 2017). The presence of the low-frequency *TET2* rs61744960 variant in MS patients of all the three families, and its frequency in unrelated MS patients cohort higher than in three databases support further investigation of this finding in relation to MS. It is of note that the SNP rs61744960 has been previously reported in an Italian study focused on leukemia, and two of the six patients, who carried this variant, had MS as a primary disease (Ottone et al., 2012).

For the *TRAF3* rs138943371 we observed in the four databases a frequency pattern similar to that of *TET2* rs61744960. *TRAF3* gene codifies for tumor necrosis factor receptor-associated factor 3, a major regulator of innate immune response through different transduction signal pathways (Cullell et al., 2017). By “Human Splicing Finder” analysis, the rs138943371 C to T change, disrupting an exonic splicing enhancer, might create in the *TRAF3* transcript a new exonic splicing silencer. Further investigation is needed to evaluate potential effects on transcription and splicing processes, that are strictly related and rely on regulatory elements in the 3′ gene region.

Although in the GWAS MS candidate genes we did not detect an increased number of low frequency variants in our MS families, those variants firstly found with parent-child transmission in MS families were detected in several combinations in unrelated patients, particularly in *C6orf10, IL2RA* and *TET2.* These observations further highlight the complexity of the MS genetic risk components.

Our study supports further investigation within genetics consortiums of multiple low-frequency risk variants in coding regions of MS candidate genes. Expression of specific protein variants, and their combinations, could provide functional insights in the heterogeneous pathogenetic mechanisms contributing to MS.

## Ethics Statement

Written informed consent was obtained from all subjects, and the study was approved by the Ethical Committee of the S. Anna University-Hospital, Ferrara, Italy.

## Author Contributions

NZ, GM, CS, MB, PZ, and FB conceived and designed the study. NZ, GM, CS, and FB wrote the manuscript. NZ, SM, and LL performed the literature revision. NZ, SM, LL, BL, NB, and IG carried out the lab experiments. NZ, CS, AB, and DB performed bio-informatic analyses of the data. FS, SS, DG, and PZ selected and recruited the patients, and performed their clinical evaluation. NZ, MB, SM, EM, and DG collected the samples and evaluated the pre-analytical variables. All authors critically evaluated the final version of the manuscript.

## Conflict of Interest Statement

The authors declare that the research was conducted in the absence of any commercial or financial relationships that could be construed as a potential conflict of interest. The reviewer RA declared a past co-authorship with the authors PZ, FB, and DG.

## Footnotes

1 http://www.ncbi.nlm.nih.gov/pubmed/

2 http://www.ebi.ac.uk/gwas

3 http://genome.ucsc.edu/

4 http://omictools.com/soapaligner-tool

5 http://www.completegenomics.com/documents/Small_Variant_Assembler_Methods.pdf

6 http://www.ncbi.nlm.nih.gov/projects/SNP/

7 http://www.1000genomes.org/data

8 http://exac.broadinstitute.org/

9 https://gnomad.broadinstitute.org/

10 http://software.broadinstitute.org/software/igv/

11 http://sift.jcvi.org/www/SIFT_enst_submit.html

12 http://www.ensembl.org/index.html

13 http://bioinfo.ut.ee/primer3-0.4.0/

14 http://www.genenetwork.org/webqtl/WebQTL.py?cmd=sch&refseq=NM_006781&species=human
