# Supplementary material for: C6orf10 Low-Frequency and Rare Variants in Italian Multiple Sclerosis Patients
Source: Front Genet. 2019 Jun 26;10:573. doi: 10.3389/fgene.2019.00573 (PMC6607989; doi:10.3389/fgene.2019.00573)
Supplement: Supplementary file 1 [file Table_1.DOCX]

**Supplementary Table 1.** List of 141 intragenic SNPs in 107 genes from MS GWAS.

| Gene | rs ID | Locus | Localization | Position (GRCh38/hg38) | References |
| --- | --- | --- | --- | --- | --- |
| *ADAMTS3* | rs78862524 | 4q13.3 | INTRONIC | 72305473 | ^1^ |
| *AGAP2* | rs12368653 | 12q14.1 | INTRONIC | 57739473 | ^2^ |
| *AGBL2* | rs7120737 | 11p11.2 | INTRONIC | 47680843 | ^3^ |
| *AHI1* | rs11154801 | 6q23.3 | INTRONIC | 135418217 | ^2, 3^ |
| *ANKRD55* | rs6859219 | 5q11.2 | INTRONIC | 56142753 | ^4^ |
| *ANKRD55* | rs71624119 | 5q11.2 | INTRONIC | 56144903 | ^3^ |
| *BACH2* | rs12212193 | 6q15 | INTRONIC | 90287050 | ^2^ |
| *BACH3* | rs72928038 | 6q15 | INTRONIC | 90267049 | ^3^ |
| *BATF* | rs2300603 | 14q24.3 | INTRONIC | 75539214 | ^2^ |
| *BTNL2* | rs4248166 | 6p21.32 | NTRONIC | 32398644 | ^5^ |
| *C1orf106* | rs7522462 | 1q32.1 | INTRONIC | 200912467 | ^2^ |
| *C1orf106* | rs55838263 | 1q32.1 | INTRONIC | 200905600 | ^3^ |
| *C6orf10* | rs3129934* | 6p21.32 | INTRONIC | 32368410 | ^6^ |
| *CBLB* | rs2028597 | 3q13.11 | INTRONIC | 105839993 | ^2^ |
| *CBLB* | rs9657904 | 3q13.11 | INTRONIC | 105867870 | ^7^ |
| *CD40* | rs4810485 | 20q13.12 | INTRONIC | 46119308 | ^3^ |
| *CD58* | rs2300747 | 1p13.1 | INTRONIC | 116561593 | ^8^ |
| *CD58* | rs1335532 | 1p13.1 | INTRONIC | 116558335 | ^2^ |
| *CD6* | rs17824933 | 11q12.2 | INTRONIC | 60993140 | ^8^ |
| *CD60* | rs6677309 | 1p13.1 | INTRONIC | 116537544 | ^3^ |
| *CD69* | rs11052877 | 12p13.31 | 3'UTR | 9753094 | ^3^ |
| *CD86* | rs9282641 | 3q13.33 | EXONIC | 122077921 | ^2^ |
| *CDH3* | rs1886700 | 16q22.1 | INTRONIC | 68652002 | ^3^ |
| *CENPO* | rs4665719 | 2p23.3 | INTRONIC | 24794991 | ^3^ |
| *CLEC16A* | rs6498168 | 16p13.13 | INTRONIC | 11141273 | ^4^ |
| *CLEC16A* | rs6498169 | 16p13.13 | INTRONIC | 11155472 | ^9^ |
| *CLEC16A* | rs11865121 | 16p13.13 | INTRONIC | 11072831 | ^8^ |
| *CLEC16A* | rs7200786 | 16p13.13 | INTRONIC | 11083944 | ^2^ |
| *CLEC16A* | rs6498160 | 16p13.13 | INTRONIC | 11105590 | ^7^ |
| *CLEC16A* | rs12927355 | 16p13.13 | INTRONIC | 11100914 | ^3^ |
| *CLECL1* | rs10466829 | 12p13.31 | INTRONIC | 9723495 | ^2^ |
| *COPB1* | rs55665837 | 11p15.2 | INTRONIC | 14473503 | ^1^ |
| *CXCR5* | rs523604 | 11q23.3 | INTRONIC | 118885029 | ^3^ |
| *CXCR5* | rs630923 | 11q23.3 | INTRONIC | 118883644 | ^2, 10^ |
| *CYP24A1* | rs2248359 | 20q13.2 | 5'UTR | 54174979 | ^2^ |
| *DDAH1* | rs11587876 | 1p22.3 | INTRONIC | 85449500 | ^3^ |
| *DKKL1* | rs2303759 | 19q13.33 | EXONIC | 49365794 | ^2^ |
| *DKKL1* | rs8107548 | 19q13.33 | INTRONIC | 49367386 | ^3^ |
| *DLEU1* | rs806349 | 13q14.2 | INTRONIC | 50285854 | ^11^ |
| *DLEU1* | rs9591325 | 13q14.2 | INTRONIC | 50237084 | ^4^ |
| *DLEU1* | rs2812197 | 13q14.2 | INTRONIC | 50243690 | ^4^ |
| *DLEU1* | rs806321 | 13q14.2 | INTRONIC | 50267187 | ^4^ |
| *DLEU1* | rs9596270 | 13q14.2 | INTRONIC | 50268304 | ^4^ |
| *ELMO1* | rs60600003 | 7p14.1 | INTRONIC | 37342861 | ^3^ |
| *EPS15L1* | rs1870071 | 19p13.11 | INTRONIC | 16394295 | ^3^ |
| *ERG* | rs2836425 | 21q22.3 | INTRONIC | 38466902 | ^4^ |
| *ETS1* | rs3809006 | 11q24.3 | INTRONIC | 128540941 | ^11^ |
| *EVI5* | rs11810217 | 1p22.1 | INTRONIC | 92682820 | ^2^ |
| *EVI5* | rs11808092 | 1p22.1 | EXONIC | 92607671 | ^5^ |
| *EVI5* | rs41286801 | 1p22.1 | 3'UTR | 92509907 | ^3^ |
| *FAM69A; RPL5* | rs6604026 | 1p22.1 | INTRONIC | 92838046 | ^12^ |
| *FCRL1* | rs2050568 | 1q23.1 | INTRONIC | 157800451 | ^3^ |
| *FLJ42102* | rs185378533 | 11q13.4 | INTRONIC | 71422087 | ^1^ |
| *FOXP1* | rs9828629 | 3p13 | INTRONIC | 71481195 | ^3^ |
| *GALC* | rs74796499 | 14q31.3 | INTRONIC | 87965984 | ^3^ |
| *GC* | rs4588 | 4q13.3 | EXONIC | 71752606 | ^1^ |
| *GEMIN2* | rs2277458 | 14q21.1 | 5’UTR | 39114277 | ^1^ |
| *GFI1* | rs6689470 | 1p22.1 | INTRONIC | 92485653 | ^4^ |
| *HAL* | rs3819817 | 12q23.1 | INTRONIC | 95984993 | ^1^ |
| *IFI30* | rs11554159 | 19p13.11 | EXONIC | 18175134 | ^3^ |
| *IFNGR2* | rs9808753 | 21q22.11 | EXONIC | 33415005 | ^11^ |
| *IL12A* | rs4680534 | 3q25.33 | INTRONIC | 159981157 | ^8^ |
| *IL12A* | rs2243123 | 3q25.33 | INTRONIC | 159991864 | ^2^ |
| *IL12A- AS1* | rs1014486 | 3q25.33 | INTRONIC | 159973324 | ^3^ |
| *IL2RA* | rs12722489 | 10p15.1 | INTRONIC | 6060049 | ^9^ |
| *IL2RA* | rs2104286 | 10p15.1 | INTRONIC | 6057082 | ^3^ |
| *IL2RA* | rs12253981 | 10p15.1 | INTRONIC | 6050383 | ^7^ |
| *IL2RA* | rs3118470 | 10p15.1 | INTRONIC | 6059750 | ^2^ |
| *IL2RA* | rs12722561 | 10p15.1 | INTRONIC | 6027930 | ^5^ |
| *IL7R* | rs6897932 | 5p13.2 | EXONIC | 35874473 | ^2^ |
| *IL7R* | rs6881706 | 5p13.2 | 3'UTR | 35879054 | ^3^ |
| *ILDR1* | rs2681424 | 3q13.33 | INTRONIC | 122050675 | ^4^ |
| *ILDR1* | rs2255214 | 3q.13.33 | INTRONIC | 122051692 | ^3^ |
| *IQCB1* | rs1920296 | 3q13.33 | INTRONIC | 121824730 | ^3^ |
| *IQGAP1* | rs8042861 | 15q26.1 | INTRONIC | 90434101 | ^3^ |
| *JAZF1* | rs9117116 | 7p14.1 | INTRONIC | 28133120 | ^3^ |
| *L3MBTL3* | rs4364506 | 6q23.1 | INTRONIC | 130068795 | ^4^ |
| *loc100506047* | rs2163226 | 2p21 | INTRONIC | 43134117 | ^3^ |
| *loc105376481* | rs1891621 | 10p11.23 | INTRONIC | 31101198 | ^4^ |
| *LPIN3* | rs6072343 | 20q12 | 3'UTR | 41339548 | ^11^ |
| *LPP* | rs4686953 | 3q26 | INTRONIC | 188365131 | ^11^ |
| *LRP2* | rs12988804 | 2q31.1 | INTRONIC | 169261301 | ^13^ |
| *MALT1* | rs7238078 | 18q21.32 | INTRONIC | 58716960 | ^2^ |
| *MANBA* | rs228614 | 4q24 | INTRONIC | 102657480 | ^2, 10^ |
| *MAPK1* | rs2283792 | 22q11.21 | INTRONIC | 21776836 | ^2, 3^ |
| *MAZ* | rs34286592 | 16p11.2 | INTRONIC | 29809159 | ^4^ |
| *MERTK* | rs17174870 | 2q13 | INTRONIC | 111907624 | ^2^ |
| *METTL1* | rs703842 | 12q14.1 | 3'UTR | 57768956 | ^12^ |
| *MMEL1* | rs3748817 | 1p36.32 | INTRONIC | 2594226 | ^3^ |
| *MPV17L2* | rs874628 | 19p13.11 | EXONIC | 18193890 | ^2^ |
| *NADSYN1* | rs4423214 | 11q13.4 | INTRONIC | 71462208 | ^1^ |
| *NCOA5* | rs2425752 | 20q13.12 | INTRONIC | 46073481 | ^2^ |
| *NDFIP1* | rs1062158 | 5q31.3 | INTRONIC | 142143435 | ^2^ |
| *NDFIP2* | rs1036207 | 5q31.3 | INTRONIC | 142119476 | ^3^ |
| *ODF3B* | rs140522 | 22q13.33 | 3'UTR | 50532837 | ^2, 11^ |
| *PDE3B* | rs116970203 | 11p15.2 | INTRONIC | 14855172 | ^1^ |
| *PHGDH* | rs666930 | 1p12 | INTRONIC | 119716347 | ^3^ |
| *PITPNM2* | rs7132277 | 12q24.31 | INTRONIC | 123108835 | ^3^ |
| *PITPNM2* | rs949143 | 12q24.31 | INTRONIC | 123110616 | ^2^ |
| *PLEKHG5* | rs3007421 | 1p36.31 | INTRONIC | 6470129 | ^3^ |
| *PVT1* | rs4410871 | 8q24.21 | INTRONIC | 127802783 | ^2^ |
| *PXT1* | rs941816 | 6p21.31 | INTRONIC | 36407527 | ^3^ |
| *RGS1* | rs7535818 | 1q31 | INTRONIC | 192575969 | ^4^ |
| *RGS14* | rs4976646 | 5q35.3 | INTRONIC | 177361569 | ^3^ |
| *RPS6KB1* | rs180515 | 17q23.1 | 3'UTR | 59946914 | ^2, 11^ |
| *RRAS2* | rs182244780 | 11p15.2 | INTRONIC | 14363985 | ^1^ |
| *SLAMF7* | rs35967351 | 1q23.3 | INTRONIC | 160742014 | ^3^ |
| *SLC2A4RG* | rs2256814 | 20q13.33 | INTRONIC | 63742630 | ^3^ |
| *SLC30A7* | rs11581062 | 1p21.2 | INTRONIC | 100941963 | ^2^ |
| *SLC44A2* | rs2288904 | 19p13.2 | EXONIC | 10631494 | ^3^ |
| *SLC9A8* | rs17785991 | 20q13.13 | INTRONIC | 49822224 | ^3^ |
| *SP140* | rs10201872 | 2q37.1 | INTRONIC | 230242009 | ^2^ |
| *SP140* | rs9989735 | 2q37.1 | INTRONIC | 230250739 | ^3^ |
| *SPON1* | rs117865811 | 11p15.2 | INTRONIC | 14180763 | ^1^ |
| *STAT3* | rs744166 | 17q21.2 | INTRONIC | 42362183 | ^14^ |
| *STAT3* | rs9891119 | 17q21.2 | INTRONIC | 42355962 | ^2^ |
| *STAT3* | rs2293152 | 17q21.2 | INTRONIC | 42329511 | ^15^ |
| *STAT3* | rs4796791 | 17q21.2 | INTRONIC | 42378745 | ^3^ |
| *STAT4* | rs996792 | 2p14.1 | INTRONIC | 191109709 | ^3^ |
| *TAGAP* | rs1738074 | 6q25.3 | 5'UTR | 159044945 | ^2^ |
| *TET2* | rs2726518 | 4q24 | INTRONIC | 105252042 | ^3^ |
| *TIMMDC1* | rs2293370 | 3q13.33 | INTRONIC | 19501087 | ^2^ |
| *TIMMDC2* | rs1131265 | 3q13.33 | EXONIC | 119503609 | ^3^ |
| *TNFRSF1A* | rs4149584 | 12p13.31 | EXONIC | 6333477 | ^8^ |
| *TNFRSF1A* | rs1800693 | 12p13.31 | INTRONIC | 6330843 | ^2, 3^ |
| *TNFSF14* | rs1077667 | 19p13.3 | INTRONIC | 6668961 | ^2, 3^ |
| *TOP3A* | rs4925166 | 17p11.2 | INTRONIC | 18307496 | ^4^ |
| *TRAF3* | rs12148050 | 14q32.32 | INTRONIC | 102797451 | ^3^ |
| *TSFM* | rs201202118 | 12q14.1 | INTRONIC | 57788279 | ^3^ |
| *TYK2* | rs34536443 | 19p13.2 | EXONIC | 10352442 | ^3^ |
| *VMP1* | rs8070345 | 17q23.1 | INTRONIC | 59739396 | ^3^ |
| *WWOX* | rs12149527 | 16q23.1 | INTRONIC | 79076699 | ^3^ |
| *ZBTB38* | rs9846396 | 3q23 | INTRONIC | 141422126 | ^11^ |
| *ZBTB46* | rs6062314 | 20q13.33 | INTRONIC | 63778360 | ^2, 11^ |
| *ZFP36L1* | rs2236262 | 14q24.1 | INTRONIC | 68794755 | ^3^ |
| *ZFP36L1* | rs4902647 | 14q24.1 | 5'UTR | 68787474 | ^2^ |
| *ZMIZ1* | rs1250540 | 10q22.3 | INTRONIC | 79276250 | ^8^ |
| *ZMIZ1* | rs1250550 | 10q22.3 | INTRONIC | 79300560 | ^2^ |
| *ZMIZ1* | rs1250542 | 10q22.3 | INTRONIC | 79274913 | ^15^ |
| *ZMIZ1* | rs1782645 | 10q22.3 | INTRONIC | 79288854 | ^3^ |
| *ZNF767P* | rs354033 | 7q36.1 | INTRONIC | 149592373 | ^2^ |

* The asterisk indicates that the rs3129934, previously assigned to the DRB1 gene, is instead located in the

C6orf10 gene.

1. Manousaki D, Dudding T, Haworth S, et al. Low-Frequency Synonymous Coding Variation in CYP2R1 Has Large Effects on Vitamin D Levels and Risk of Multiple Sclerosis. Am J Hum Genet 2017;101:227-238.

2. International Multiple Sclerosis Genetics C, Wellcome Trust Case Control C, Sawcer S, et al. Genetic risk and a primary role for cell-mediated immune mechanisms in multiple sclerosis. Nature 2011;476:214-219.

3. International Multiple Sclerosis Genetics C, Beecham AH, Patsopoulos NA, et al. Analysis of immune-related loci identifies 48 new susceptibility variants for multiple sclerosis. Nat Genet 2013;45:1353-1360.

4. Andlauer TF, Buck D, Antony G, et al. Novel multiple sclerosis susceptibility loci implicated in epigenetic regulation. Sci Adv 2016;2:e1501678.

5. Zhou Y, Zhu G, Charlesworth JC, et al. Genetic loci for Epstein-Barr virus nuclear antigen-1 are associated with risk of multiple sclerosis. Mult Scler 2016;22:1655-1664.

6. Comabella M, Craig DW, Camina-Tato M, et al. Identification of a novel risk locus for multiple sclerosis at 13q31.3 by a pooled genome-wide scan of 500,000 single nucleotide polymorphisms. PLoS One 2008;3:e3490.

7. Steri M, Orru V, Idda ML, et al. Overexpression of the Cytokine BAFF and Autoimmunity Risk. N Engl J Med 2017;376:1615-1626.

8. De Jager PL, Jia X, Wang J, et al. Meta-analysis of genome scans and replication identify CD6, IRF8 and TNFRSF1A as new multiple sclerosis susceptibility loci. Nat Genet 2009;41:776-782.

9. International Multiple Sclerosis Genetics C, Hafler DA, Compston A, et al. Risk alleles for multiple sclerosis identified by a genomewide study. N Engl J Med 2007;357:851-862.

10. International Multiple Sclerosis Genetics C, Lill CM, Schjeide BM, et al. MANBA, CXCR5, SOX8, RPS6KB1 and ZBTB46 are genetic risk loci for multiple sclerosis. Brain 2013;136:1778-1782.

11. Lill CM, Luessi F, Alcina A, et al. Genome-wide significant association with seven novel multiple sclerosis risk loci. J Med Genet 2015;52:848-855.

12. Australia, New Zealand Multiple Sclerosis Genetics C. Genome-wide association study identifies new multiple sclerosis susceptibility loci on chromosomes 12 and 20. Nat Genet 2009;41:824-828.

13. Zhou Y, Graves JS, Simpson S, Jr., et al. Genetic variation in the gene LRP2 increases relapse risk in multiple sclerosis. J Neurol Neurosurg Psychiatry 2017;88:864-868.

14. Jakkula E, Leppa V, Sulonen AM, et al. Genome-wide association study in a high-risk isolate for multiple sclerosis reveals associated variants in STAT3 gene. Am J Hum Genet 2010;86:285-291.

15. Patsopoulos NA, Bayer Pharma MSGWG, Steering Committees of Studies Evaluating I-b, et al. Genome-wide meta-analysis identifies novel multiple sclerosis susceptibility loci. Ann Neurol 2011;70:897-912.
