# Supplementary material for: C6orf10 Low-Frequency and Rare Variants in Italian Multiple Sclerosis Patients
Source: Front Genet. 2019 Jun 26;10:573. doi: 10.3389/fgene.2019.00573 (PMC6607989; doi:10.3389/fgene.2019.00573)
Supplement: Supplementary file 2 [file Table_2.DOCX]

**Supplementary Table 2.** Combination of the low-frequency variants in *C6orf10* with WES variants within the unrelated MS patients.

| ID | Gender | Age of  MS onset | Phenotype  at examination | *C6orf10* | *CD86* rs11575853 | *EVI5* rs41286809 | *GC* rs76781122 | *IL2RA* rs12722600 | *MALT1* rs74847855 | *TET2* rs61744960 | *WWOX* rs7201683 |
| --- | --- | --- | --- | --- | --- | --- | --- | --- | --- | --- | --- |
| 138 ZM | F | 21 | RR | **Ser454Xfr (Het)** |  |  |  |  |  |  |  |
| 221 ZM | M | 22 | RR | Val559Leu (Het) Glu561Asp (Het) |  |  | Het |  |  |  |  |
| 150 ZM | F | 25 | RR | **Ser389Xfr (Hom)** |  |  |  |  |  |  |  |
| 194 ZM | F | 31 | RR | Gln385Glu (Het) Thr429Ser (Het) Gly477Val (Het) |  |  | Het | Hom |  |  | Het |
| 106 ZM | M | 32 | SP | **Asp504Val (Het)** **Asp522Asp (Het)** |  |  |  |  |  |  |  |
| 128 ZM | F | 32 | RR | **Glu506Val (Het)** |  |  |  |  |  |  |  |
| 109 ZM | F | 37 | SP | **Asp522Asp (Het)** | Het | Het |  |  |  |  |  |
| 115 ZM | F | 38 | SP | **Lys457stop (Het)** |  |  |  |  | Het | Het | Het |
| MS23 | F | 38 | RR | **Gly451stop (Het)** | Het |  |  |  |  |  |  |
| 112 ZM | F | 41 | RR | Lys557Ile (Het) |  |  |  | Het |  |  |  |
| 25-WP3 | F | 51 | PP | **Asp516Tyr (Het)** |  |  |  |  |  |  |  |
| 65-WP3 | F | 51 | SP | **Asp522Asp (Het)** |  |  |  | Het |  |  |  |

The heterozygous (Het) or homozygous (Hom) condition of the variants is specified.

In bold and black, the *C6orf10* stop gained and frame shift variants are highlighted. In bold and gray, the *C6orf10* variants that were in repetitive regions.
