# Supplementary material for: C6orf10 Low-Frequency and Rare Variants in Italian Multiple Sclerosis Patients
Source: Front Genet. 2019 Jun 26;10:573. doi: 10.3389/fgene.2019.00573 (PMC6607989; doi:10.3389/fgene.2019.00573)
Supplement: Supplementary file 3 [file Table_3.DOCX]

**Supplementary Table 3** - LD analysis of C6ORF10 and the most significant MS GWAS SNPs

| **Gene** | **SNP** | **Localization** | **Position**  **(Ch37/hg19)** | **Distance Kb** | **Coefficient of correlation (*r*^2^)** | **Disequilibrium coefficient (*D*')** |
| --- | --- | --- | --- | --- | --- | --- |
| *C6ORF10* | rs16870005 | Exonic | 32261153 |  |  |  |
| *C6ORF10* | rs3129934 | Intronic | 32336187 | 75.034 | 0.112559 | 0.999998 |
| ***Tag SNPs for HLA-DRB1*15:01*** | | | | | | |
| *HLA-DRA* | rs3135388 | Downstream-gene | 32413051 | 151.898 | n.a | n.a |
| *HLA-DRA* | rs3129889 | Downstream-gene | 32413545 | 152.392 | n.a. | n.a. |
| *HLA-DRB1 - HLA-DQA1* | rs9271100 | Intergenic | 32[576478](http://grch37.ensembl.org/Homo_sapiens/Location/View?contigviewbottom=variation_feature_variation%3Dnormal%2Cseq%3Dnormal;db=core;r=6:32576428-32576528;source=dbSNP;v=rs9271100;vdb=variation;vf=263078675) | 315.325 | 0.053394 | 0.999980 |
| *HLA-DRB1 - HLA-DQA1* | rs9271366 | Intergenic | 32586854 | 325.701 | 0.054751 | 0.999990 |
| *HLA-DQA1* | rs2040406 | Upstream-gene | 32603007 | 341.854 | n.a. | n.a. |

LD analysis of the low frequency rs16870005 with: 1) the GWAS intronic rs3129934 within C6ORF10; 2) the tag SNPs for HLA-DRB1*15:01 rs3135388, rs3129889, rs9271366 and rs2040406, with the most significant MS GWAS association as previously indicated.

n.a. = LD estimate not available. Note: a variant may have no LD data in a given population for the following reasons: i) estimated r^2^ values below 0.05 and thus filtered out; ii) minor allele frequency close or equal to 0 (this is not the case for these SNPs that showed a MAF > 0.084 in CEU); iii) not enough genotypes to calculate LD values (the number of genotypes available range from 2687 – 4132).
